# Virtue and austerity

**DOI:** 10.1111/j.1466-769X.2012.00550.x

**Published:** 2012-12-20

**Authors:** Peter Allmark

**Affiliations:** Principal Research Fellow, Health and Social Care Research Centre, Sheffield Hallam UniversitySheffield, UK

**Keywords:** ethics, human flourishing, moral theory, virtues, politics, Aristotle, situationist psychology, John Doris

## Abstract

Virtue ethics is often proposed as a third way in health-care ethics, that while consequentialism and deontology focus on action guidelines, virtue focuses on character; all three aim to help agents discern morally right action although virtue seems to have least to contribute to political issues, such as austerity. I claim: (1) This is a bad way to characterize virtue ethics. The 20th century renaissance of virtue ethics was first proposed as a response to the difficulty of making sense of ‘moral rightness’ outside a religious context. For Aristotle the right action is that which is practically best; that means best for the agent in order to live a flourishing life. There are no moral considerations besides this. (2) Properly characterized, virtue ethics can contribute to discussion of austerity. A criticism of virtue ethics is that fixed characteristics seem a bad idea in ever-changing environments; perhaps we should be generous in prosperity, selfish in austerity. Furthermore, empirical evidence suggests that people indeed do change with their environment. However, I argue that virtues concern fixed values not fixed behaviour; the values underlying virtue allow for different behaviour in different circumstances: in austerity, virtues still give the agent the best chance of flourishing. Two questions arise. (a) In austere environments might not injustice help an individual flourish by, say, obtaining material goods? No, because unjust acts undermine the type of society the agent needs for flourishing. (b) What good is virtue to those lacking the other means to flourish? The notion of degrees of flourishing shows that most people would benefit somewhat from virtue. However, in extreme circumstances virtue might harm rather than benefit the agent: such circumstances are to be avoided; virtue ethics thus has a political agenda to enable flourishing. This requires justice, *a fortiori* when in austerity.

## Introduction

In the literature on health-care ethics, the virtue approach is sometimes presented as a third way between consequentialism and deontology, the latter approaches focusing on the act, the virtue approach on the agent. Implied is that all three share the same goal, to help the professional do the morally right thing. Thus characterized the virtue approach would seem to have least of the three to say about austerity as this raises issues about justice and distribution, not character. In this paper I make two claims that: (1) this is a poor representation of the virtue approach and (2) austerity raises issues to which a genuine virtue approach can make useful contributions. The paper is presented in two main sections based on the claims.

## Claim 1 – virtue ethics is not a third way in moral philosophy

The reappearance of virtue in moral philosophy is usually traced back to the paper ‘Modern Moral Philosophy’ by Anscombe (1958). Her call for a return to the approach found in Aristotle and Plato was taken up in the theoretical realm by *inter alia*Foot (1978), Geach (1977), Irwin (1990) and Macintyre (2007), and in the practical realm of health-care ethics by many others (Hursthouse, 1987; Gardiner, 2003; Bolsin *et al*., 2005; Armstrong, 2006; Hodkinson, 2008). In the practical realm, the task of virtue ethics is taken to be that of other approaches, such as consequentialism and deontology, that is, to help practitioners with difficult ethical decisions in practice, such as abortion, truth telling, and whistleblowing. In this task, virtue is taken as providing either an alternative approach (Hursthouse, 1987; Armstrong, 2006) or a complementary one (Beauchamp & Childress, 2008). In the first, the virtues enable the agent to discern the right action while other theories do not help; in the second, the virtues enable the agent correctly to apply the other theories. Both versions appeal to some nurse theorists as they seem to imply the virtuous agent has a tacit or intuitive expertise (practical wisdom) similar to that which the expert nurse is said to have in, for example, Benner's approach (Benner, 1997, 2000).

Viewed as this third way in ethics, the virtue approach, particularly the alternative account, seems circular; the right action is what the virtuous agent would do, and the virtuous agent would do the right action. And in discussion of practical issues, this circularity manifests as it-depends answers: is abortion wrong – it depends; is lying to patients wrong – it depends; and so on. The practitioner is referred back to what the virtuous agent would do, and that cannot be inferred outside the specific situation. In an article for this journal, Holland expresses well the frustration of the practitioner; any nurse who defended a particular act on the basis either that she has years of experience or intuitively knows what is right would get short shrift (Holland, 2010).

This is not to decry the notions of expertise, intuition, and practical wisdom. An art expert might be able instantly to recognize a fake; an art buyer would do well to follow her advice. But at the heart of this advice is an agreed definition of the work as either fake or not. And although the expert made her judgement instantaneously and may credit her intuition, asked to give reasons for the judgement she could provide them. This is unlike the situation with issues in health-care ethics. There is no widely agreed definition of moral rightness and *a fortiori* no agreement on who is expert in judging it. Faced with practical problems in health-care ethics the practitioner can draw upon act-based approaches that at least provide some equipment with which to make judgements; in contrast, the virtue approach, and other character-based approaches, such as the ethics of care, offer the will-o-the-wisp person who somehow knows.

The temptation to characterize virtue ethics as this third type of moral philosophy is reinforced by the fact that for modern readers the terms ‘virtue’ and, particularly ‘moral virtue’ (the usual translations for Aristotle's αρετή and αρετή εθική) imply moral goodness. Reading Aristotle this way it seems that he has fixed it so that magically it turns out that the good life for all is also and only the life of a morally good human; doing well requires being good. This conclusion runs against the apparently obvious fact that,

Some people live in hell, many bastards succeed (Michael Gira: lyrics to ‘Failure’ by The Swans)

So there are two issues here: first, why Aristotle seems to conflate living a morally good life with a personally flourishing one and, second, how this account can survive in the face of the fact that virtues are not always and perhaps not often associated with a life we could describe as flourishing, happy or any such thing.

Tackling the first issue takes us back to Anscombe and those who followed, particularly Macintyre and Foot (Foot, 1978; MacIntyre, 2007). In brief, the story they tell is of Aristotelian and Stoic concepts of virtue being adopted in the post-Roman world by the nascent Christian one. Here they became grounded not in the notion of individual flourishing but in that of law-like injunctions given by God; doing the right thing became not right-for-me but, rather, right-by-God. This neatly resolved a central problem for the classical philosophers: how to incorporate justice into the virtue account of living well. Justice had been condemned by Glaucon as good for another:

[No] man thinks justice pays him personally, since he will always do wrong when he gets the chance. Plato *Republic* 360c. (Plato, 1987)

In response, Plato, Aristotle, and others struggled to fit acting in accord with justice into a personally flourishing life; the idea that being unjust to another damaged the unjust person more than the wronged one seemed as strange to many Greeks as to us. On the Christian account, justice is one of God's injunctions to us; the rewards for justice, and the final righting of wrongs, come after death. The apparent flourishing of the unjust in the here-and-now is of little matter in the face of eternal damnation or reward.

What Macintyre terms ‘the Enlightenment project’ is the attempt to give these law-like injunctions a secular basis; the paradigm example, Kant, terms the injunctions of law-like ethics the ‘categorical imperative’ and attempts to show that they can be derived from reason. Anscombe's starting point is that this project has failed; we have inherited law-like morality but have failed to find a non-religious grounding for it. Her call for a return to the ethics of Aristotle is not in the belief that this will complete the Enlightenment project but rather that it will replace it with a politics and ethics based on the idea of human flourishing. Thus, the reason the *Nicomachean Ethics* can seem to us like a book of moral philosophy, an attempt to complete the Enlightenment project, is because the language of virtue has been absorbed into the language of law-like morality. To make sense of Aristotle's ethics we must distil the Aristotelian essence from this Enlightenment brew:

It is important that our account of flourishing and good fortune be uncontaminated with contemporary thoughts of morality. (Coope, 2006, p. 24)

Aristotle's *Nicomachean Ethics* is more like a work of modern self-help than modern moral philosophy: *How to live well* not *How to be good*. His work that has subsequently been termed ‘ethics’ and ‘politics’ he terms the ‘philosophy of human affairs’ (NE 1181b). Linked to his other work, such as his biology, Aristotle is providing an account of what it is for creatures like us to live well, to live successfully. In brief, his answer is that we need the virtues, such as temperance and courage, and we also need some good fortune to avoid external events such as disease, earthquakes, and wars, that can damage or destroy a flourishing life. Good fortune is also needed to give the individual a chance of developing virtue in the first place – both the circumstances of birth and the potentials with which people are born are outside their control.

Having set out the argument for the claim that Aristotle's virtue ethics should not be seen as a third type of moral philosophy, we are faced with the second issue, the thought that Aristotle's belief in human flourishing consisting of acting virtuously is implausible. We shall tackle this issue through developing the second main claim of this paper, that austerity raises issues to which a genuine virtue approach can make useful contributions.

## Claim 2 – austerity raises issues to which virtue ethics can make a useful contribution

In the wet, cacti wilt and fungi flourish. For any species an austere environment is roughly one in which some of its members can survive but few can flourish. Some species are ill-equipped to survive in any but the most specific of circumstances; for these, such as Giant Pandas, the environment is either conducive to flourishing or positively hostile – there are no grades of austerity. By contrast, humans are adaptable; we can survive in many environments. Of these, some are more or less austere, a few flourish or many flourish. And this raises the issue we touched upon at the end of the previous section. Virtues are firm states of character; for example, someone who is courageous will always be so. And, claims Aristotle, those with virtues are most likely to flourish. The experience of austerity, however, seems to show otherwise; to flourish, humans need to be adaptable.

Furthermore, humans are indeed adaptable: generous when they can afford to be; kind when it is easy; selfish when required. John Doris argues that it is the environment that primarily determines our choices, not our character (Doris, 2002). Numerous experiments in social psychology show this. To take one amusing example, students on a seminary for study of religious education were told to give a short talk, half of them on the story of the Good Samaritan, the other half on a topic unrelated to helping (Darley & Batson, 1973). They were set up so that on their way they would come upon someone needing help, a man slumped in an alleyway. Some of the students were told they were early, some, just on time, others, that they were late. The likelihood of stopping to help correlated along these lines; the early students stopped to help, the late ones did not. The topic of the proposed talk made no difference. Thus overall, it is said, not only is the link between virtue and flourishing implausible, but also, human behaviour reflects this. In different environments people should behave differently in order to flourish, and in fact they do. How might an Aristotelian respond?

One problem with Doris's critique is that he fixes behaviour too tightly to character types, such as courage. In the Good Samaritan experiment, he takes it that those in a hurry who pass by do not have the virtue of charity and, therefore, it is likely that those who have plenty of time and who stop probably do not have it either. But it is not obvious that possession of the virtue of charity would result in someone's stopping to help. Those who pass by might feel a strong sense of duty^1^ to deliver the lecture to the waiting students. While they would stop to save someone's life, they would, under pressure of time, pass by someone who may simply be drunk. Virtue is not evinced by particular action types but rather in the values underlying it, the reason the action is chosen. It is not an act of charity to disappoint the waiting congregation unless the cause is worthwhile. In this example, the students may have judged it was not.

To threaten the virtue account of flourishing, Doris would need to show, first, that people do not have or act upon established values and, second, that people would not do well to have or act upon certain (virtuous) established values. He does not do this; indeed, his commentary on the experiments reveals a belief that people are consistent in one respect, that they do what they perceive to be in their best interest; stealing when able to get away with it, and so forth. Aristotle would agree that this is what people do*.* He would not accept, however, that people's vision of their own best interest is always correct. The virtuous agent has a consistent vision of her best interest from which her behaviour reliably follows; called upon to risk her well-being in a worthwhile cause, she will do so. In other circumstances she will not risk her well-being. Doris falsely infers that this irregularity in behaviour (taking risks only sometimes) shows the agent to be inconsistent whereas, in fact, she is consistent in her vision and values and, therefore, her action.

This takes us part way to rebutting the criticism that people need to be adaptable if they are to flourish. We can say that consistent values will result in different choices for different situations: for example, taking a risk in one situation but not another. But there is further to go. Recall that we are now operating within an Aristotelian practical philosophy concerned not with moral rightness but rather rightness in relation to human flourishing. Even on the account given so far, which allows for adaptable and flexible behaviour grounded in stable values, the problem remains that non-virtuous agents seem to flourish. This problem has two aspects. The first is that Aristotle put justice to the fore; in our relations with others, justice is

complete virtue … For this reason, it is often held that justice is the greatest of the virtue, and that ‘neither evening star nor morning star is such a wonder’ (Aristotle, 2000) (1129b).

But because it concerns our relationship with others more obviously than, say, temperance and courage, it is far from clear that justice serves the individual well. The second aspect is that fate seems to play a large role in flourishing such that many people would not flourish even if they were virtuous.

One way round both aspects of the problem is to say that common ideas of flourishing are almost completely wrong. This is the route taken by the Stoics. Like most classical writers on ethics and flourishing the Stoics were much exercised with the problem of fate (Annas, 1995). In our common ideas, flourishing can be undermined even late in life. Someone who appears happy, comfortable, and virtuous might be undermined by illness (her own or in those she loves), a child going ‘off the rails’, even a house fire. The Stoics give an account of flourishing in which it can be attributed only to someone who has rendered himself untouchable by fate. The Stoic virtuous agent meets good fortune or bad with the same, Stoic, indifference; he would be happy even being tortured (NE 1153b). This indifferent state they termed ataraxia; it has been compared to the state aimed at through Buddhist meditation (Coseru, 2007). While this account successfully navigates the problem of fate, it does so at the expense of being unlike anything most would recognize as a flourishing life. In particular, being indifferent seems to take away much possibility of enjoying life, and enjoyment seems to be crucially important for a flourishing life. For example, parents’ pleasure in the development of their children is based in caring deeply for them; this leaves them exposed to terrible pain if things go wrong; but without such deep caring, their lives would look hollow to most observers. This is illustrated in the alleged (but most likely untrue) response of a Stoic father, Anaxagoras, to news of his son's death,

I was aware that it was a mortal I had begotten. (Freeman, 1935, p. 73)

By contrast, Aristotle's account of flourishing is recognizable and worldly. And most of the virtues of intellect (such as intelligence) and character (such as courage) are plausibly helpful to live a good life. It seems that in most circumstances a clever and courageous person will fare better than someone lacking intelligence or racked by anxiety. Aristotle's account is also recognizable in that he takes it that a flourishing life is one that is pleasurable or enjoyable. His account, however, is susceptible to the problems of justice and fate.

As stated above, the problem of justice seeming to be another person's good rather than that of the just individual was well known to Aristotle. Aristotelians writing since Anscombe's paper have often given a naturalist account of virtue and flourishing in which justice is more a virtue for the species than the individual (MacIntyre, 1999; Foot, 2001). As social, rational, dependent animals, justice gives all humans in a society the best chance of flourishing. The problem of an individual benefiting from being unjust can thus be seen as a version of the prisoner's dilemma; we would all do best to be just but if others are unjust it might serve me best to be unjust too. Thus in terms of my individual best interest the reason I have to be just is a collective one: I benefit if I live in a society in which justice prevails; my acting justly contributes to this; therefore I do well to act justly.

At first glance, this response to the problem of justice seems not watertight; in an otherwise wholly just society might not the unjust person flourish? However, Aristotle's oft-cited belief (from *Politics* 1252b30) that man is a social or political animal is important here. It means that part of his answer to the old question, ‘How should I live?’ is ‘… in a flourishing society’. In order for such a society to prevail, justice must prevail. Unjust acts undermine this and thus undermine the individual's own flourishing. This is not merely because of abstract sympathy for those suffering injustice. As social animals we have concern for others who also rely on justice: family, friends, and so on. An unjust act might help us, say, obtain material goods but it threatens the well-being of those we care about and therefore ours (Buckle, 2002). If the unjust person has no concerns for others then he is not flourishing as a social being anyway.

Turning to the problem of fate, it manifests in different ways: people can be rendered unable to live flourishing lives by *inter alia* illness, lacking sufficient intelligence, living in an unjust society, living without sufficient material wealth. Arius Didymus provides a useful tripartite categorization of goods here: goods of the body and of the soul, and external goods (Didymus & Simpson, 2010). The goods of the soul are the moral (the term here denoting character) and intellectual virtues, the goods of the body are physical virtues, such as strength and health, and external goods are such things as good rulers and material wealth. In Aristotelian style he defines flourishing or happiness as virtuous activity ‘furnished with the equipment you would pray for’. He goes on,

[The] virtuous man would also use virtue well amid evils, though he will not be blest, and since he would be displaying his noble breeding amid injuries, though he be not happy … [happiness] does not mean bearing up amid things terrible but enjoying good things along with preserving justice in community as well and not depriving itself of the beauties of study or of the necessities of life. [132.8]

Arius thus gives us a variety of ways in which we can fall short of flourishing: lacking goods of the body, of the soul, external things, or a combination. For argument's sake here we shall group body and external goods together as ‘external’. A virtuous person can be ill, ugly, and poor; a vicious one, healthy, handsome, and rich. Both, it would seem, are in a state below complete flourishing and happiness but above that of complete destitution of body and soul; they are in an intermediate state. What reason has either to remain or become virtuous? Perhaps the virtuous agent should give up on, say, justice, and take to stealing to supplement his external goods. Thuswise he might lose his goods of the soul but get in return external goods. And the vicious agent is already in that position; what has he to gain from virtue, particularly if his vices are what got him external goods such as wealth in the first place?

In a naturalist account, our response to these questions can be partly empirical. We see examples of good people whose lives are blighted with misfortune, whose goodness has enabled them to maintain their sense of dignity, worth, pride, and self-esteem. It might also help people keep their sanity. I do not seek to break the link between enjoyment and flourishing here; rather I take it that such things as dignity and worth result from or consist in enjoyment or pleasure that can provide some compensation for misfortune.^2^ Of non-virtuous agents blessed with good fortune, we might suggest that lacking goods of the soul risks the other goods; the clearest example is the way intemperance harms health. Although less obvious, I have suggested also that injustice harms the unjust person because it undermines the social fabric on which his well-being depends *qua* rational social animal.

This gives us an outline account of why unfortunate virtuous people should remain virtuous and why fortunate non-virtuous people risk losing their intermediate happiness. There remains the question of what people should do in environments in which vice improves your chance of being in the intermediate state rather than in a state of total destitution or death. Although the vicious agent in a bad environment will not flourish, he will fare better than the virtuous one.

One response to this would be to ask whether such environments are likely to be common. It would need to be one in which clever and talented people would do better to cultivate vice than virtue – given vice's potential to harm personal health and friendship this seems counterintuitive. But granted its possibility what this amounts to is saying that some environments are so bad for humans that flourishing is out of the question and the best chance of survival lies in the development of traits that run against rational, social nature as, for example, selfishness does. An Aristotelian should grant this possibility. One implication is that individuals should act in order to avoid or to alter such systems; Aristotle's ethics thus has political implications.

I began this section with the claim that austerity raises issues to which virtue ethics can make a useful contribution. These issues concern the relationship between virtue, the environment, and human flourishing. I offered a rebuttal of the criticism that flexibility rather than virtue is required for humans to flourish in changing environments. From this basis I suggested that some environments are more or less conducive to flourishing. Austere environments are relatively non-conducive. We tend to think of austerity in economic terms, but a child growing up in an environment that is wealthy but short of parental love faces austerity. Thus it is reasonable to talk of degrees of flourishing in people's lives; the child without parental love may go on to flourish but it would have been better had he known love when young; people in prosperous but acquisitive societies will find it hard to develop the virtue of justice and thus to flourish. Similarly, illness is never welcome but possession of virtue enables people to fare better in its presence. However, certain environments make flourishing impossible. Severe mental illness provides an example, living in a terribly unjust society another. In some such cases the individual might be better served by vice rather than virtue: for example, willing to sacrifice others to survive. Such situations are wholly unfortunate and to be avoided if humans are to flourish.

## Conclusion

In years of abundance, most of the young people have the wherewithal to be good, whereas in years of adversity, most of them become violent. This is not a matter of a difference in the capacities sent down by Heaven but rather of what overwhelms their minds. Mengzi. (Chinese Confucian Philosopher, 4th century BC, cited in Bloom, 2002, p. 83)

Virtue ethics can sound priggish and absurd when used as a form of Enlightenment moral philosophy. We are to emulate super-humans who both know what is morally right and desire above everything to bring it about. Stripped of this, however, it is a practical philosophy with political implications (Buckle, 2002). Aristotle makes clear that his Ethics is part of an overall practical philosophy that includes politics. Humans are social animals; it follows that an account of human flourishing is also an account of social flourishing. The point Mengzi makes (above) is that people make environments and are made by them; if the environment is hostile to flourishing, agents are unlikely to develop the personal characteristics needed or the social environment needed for flourishing.

In this regard, recent work by Nussbaum and others is useful (Nussbaum, 2006). This grounds the language of human rights in flourishing as individuals and as societies; we have the right to live the best life possible with the capacities we have as individuals and the resources we have as a society; social systems are just to the extent that they enable this. To this point, approaches such as Nussbaum's have primarily been applied to broad political concerns, such as famine, rather than to the specific area of health-care decision making; but the field is opening up to include application to health and health-care ethics (Anand, 2005; Mitra, 2006; Coast *et al*., 2008). As practitioners in health care, we would do well to view health-care ethics as enmeshed with a concern with flourishing rather than morality.

## Footnotes

1 The term ‘Duty’ sounds law-like and out of line with the argument in the previous section. Buckle (2002) shows how the notion of law is compatible with and central to the account of ethics and politics based on flourishing. I ask the reader to accept this *ad hominem*.

2 Diane Lamplugh, mother of the (presumed) murdered estate agent Suzy Lamplugh, went on to become an expert in the issue of people who disappear and to found the charity, the Suzy Lamplugh Trust, which helped set up guidelines on lone-working with which many health workers are familiar. By all accounts, she was a good person both before and after her daughter disappeared. She died on 18 August 2011. Unfortunately, it seems wrong to describe her life as happy or flourishing but her charitable work plausibly provided some compensation.
